# The Impact of a Commercial Electrolyte Beverage on the Hydration Status of Active Men and Women

**DOI:** 10.3390/nu17030585

**Published:** 2025-02-05

**Authors:** Cary Boyd-Shiwarski, Evan Ray, Harikesh Subramanian, Nicole Zharichenko, Amy Monroe, Aman Mahajan

**Affiliations:** 1 Department of Medicine, Renal and Electrolyte Division, University of Pittsburgh, Pittsburgh, PA 15260, USA; 2 Department of Anesthesiology and Perioperative Medicine, University of Pittsburgh, University of Pittsburgh Medical Center (UPMC), Pittsburgh, PA 15260, USA

**Keywords:** electrolytes, renal, supplement, dehydration, body hydration index

## Abstract

Background/Objectives: Hypo-hydration is a major health concern that affects performance and is associated with increasing morbidity and growing health care costs. There is an emerging interest in optimizing hydration and identifying how factors such as ingestion rate and beverage composition affect hydration. This study examined three beverages with varying ingestion rates and measured markers of hydration. Methods: Thirty healthy, active participants between the ages of 18 and 45 years were given three different beverages on three separate days. The beverages were of identical volumes (1 L), but differed in the rate of ingestion, carbohydrate content and electrolyte content. Beverage 1 and water alone were both consumed at a metered rate of one liter over four hours, whereas Beverage 2 was used as a positive control and was consumed at a bolus rate of one liter in 30 min. Results: After six hours, Beverage 1 significantly improved markers of hydration compared to water alone or Beverage 2. Beverage 1 decreased cumulative urine output vs. water alone by 32% (absolute difference −0.33 L; CI ± −0.16 to −0.51) and vs. Beverage 2 by 26% (absolute difference −0.26 L; CI ± −0.13 to −0.38). Beverage 1 increased the beverage hydration index vs. water alone by 64% (absolute difference +0.64 L; CI ± 0.36 to 0.92) and vs. Beverage 2 by 48% (absolute difference +0.53 L; CI ± 0.30 to 0.76). Conclusions: Beverage 1 is superior to water alone at improving hydration when it is ingested at similar rates. Moreover, metered ingestion of Beverage 1 improved hydration compared to a bolus ingestion of Beverage 2, this could be due to the dissimilar ingestion rates and/or beverage composition.

## 1. Introduction

Preserving hydration through renal handling of water and electrolytes is pivotal to survival [1,2,3]. Euhydration refers to a state of “normal” body water content (with changes less than ±0.2–0.5% of total body water), with “hypo- and hyperhydration” being either a water deficit or excess beyond these limits—and the term “dehydration” refers to a 3% or greater loss of total body water producing hypertonicity [2,4,5]. Dehydration can result in renal failure, arrhythmia, fainting, headache, fatigue, seizures and mental status changes [6]. Identifying factors and/or supplements that improve hydration compared to water alone can play an important role in preventing severe hypohydration and dehydration.

Studies have shown that exercising athletes rarely ingest enough fluid to match their sweat loss, with some studies suggesting fluid intake typically compensates for only 50% of sweat loss during physical activity [7,8,9,10]. Athletes are not the only population vulnerable to hydration deficits. Those that work in hot environments, the elderly, those with increased gastrointestinal output (i.e., vomiting, diarrhea, ostomy output, inflammatory bowel disease), poor oral intake, pregnancies complicated with hyperemesis gravidarum, peri-operative patients, and patients with long term health conditions including cancer, diabetes and alcohol use can all be affected by hypo-hydration. It has been estimated that over half a million hospitalizations per year are due to dehydration with a cost of over USD 5.5 billion [11]. Thus, there are both practical and clinical reasons to identify simple, cost-effective methods to promote euhydration.

Factors that influence hydration include beverage volume, timing and composition. It is well-established that plain water alone is ineffective for restoring hydration, as it lowers plasma osmolality and induces diuresis [12]. Rapid ingestion can also trigger diuresis due to the protective bolus response, which inhibits the release of arginine vasopressin (AVP) [13]. Slower metered rates of ingestion are thought to improve hydration compared to rapid bolus consumption [5,14,15,16]. Adding electrolytes (i.e., sodium Na^+^, potassium K^+^, chloride Cl^−^) and macronutrients (carbohydrates, fat, protein) can help delay diuresis by altering gastric emptying, intestinal absorption, and kidney excretion [17]. While several studies suggest that increased electrolyte content is the most critical factor to improve hydration, further research is needed to understand how the balance of electrolytes to macronutrients, as well as the rate of ingestion, impact hydration [5,17,18,19,20,21,22,23,24].

Despite the overwhelming number of commercial hydration beverages on the market, there are only a limited number of studies that address whether these beverages are effective at improving hydration. The purpose of this study was to determine whether Beverage 1, a commercial hydration beverage, improved markers of hydration compared to plain water. Beverage 1 is an unflavored, sugar-free, and sweetener-free electrolyte supplement. According to the manufacturer’s nutrition label, it is primarily made up of electrolytes including Na^+^, Cl^−^ and K^+^ (Table 1). It can be added dropwise to any beverage to increase the beverages’ electrolyte content and is designed to be consumed in small aliquots in beverages throughout the day and has been purported to prevent hypohydration and electrolyte loss. This study is a comparison between Beverage 1 and plain water. This study also included another commercially available hydration product (Beverage 2), as a positive control as it has been previously published to improve markers of hydration [25]. For this study Beverage 1 and 2 contained similar amounts of sodium (600 mg) but differed in other electrolyte and macronutrient content (Table 1). The primary aim of this study was to determine whether Beverage 1 improved hydration compared to water alone, and the secondary aim was to measure whether there were any hydration differences between Beverage 1 and Beverage 2.

## 2. Materials and Methods

Ethics Approval: This is a single-center prospective, cross-over, placebo-controlled clinical trial at the University of Pittsburgh Medical Center (UPMC). All experimental procedures were approved by the University of Pittsburgh Medical Center Institutional Review Board approval number STUDY22090018 and consent was approved for eligible patients. The trial was registered at www.clinicaltrial.gov under NCT05768789. Participants completed a health history and informed consent prior to the start of the study.

Study Population: This study enrolled adult participants who were healthy volunteers, and were defined as active based on their ability to walk up at least one flight of stairs without difficulty. The participants were at rest for the duration of the study. The inclusion criteria included any male or female patient ≥18 to 45 years of age, non-tobacco users, negative pregnancy test in women of childbearing potential, creatinine ≤ 1.2 milligram/deciliter (mg/dL), no known underlying medical conditions, willingness to refrain from alcohol for 24 h prior to testing day, willingness to refrain from strenuous exercise for 24 h prior to each test day, without any obvious signs or symptoms of infection. Patients who were excluded had a creatinine lab value > 1.2 mg/dL, proteinuria/hematuria/glucosuria based on urine dipstick, diagnosed medical condition that would impede results (congestive heart failure, hypertension, coronary artery disease, chronic kidney disease, history of electrolyte abnormality), pregnancy, use of diuretics within two weeks prior to first day of trial, active infection based on symptoms (bacterial or viral), hemodynamic abnormality at screening visit with blood pressure less than 100/60 mm of mercury (mmHg) or greater than 140/90 mmHg. While similar prior studies did not set blood pressure cutoffs, we chose to minimize variability among participants by excluding those with extremely high or low blood pressures, as such conditions could affect hormone levels involved in water regulation [26]. Additionally, participants with overt stage 2 hypertension were excluded due to concerns that increased sodium intake could exacerbate hypertension [27]. Prior to initiation of the study, participants completed informed consent and a health history. During the initial visit female participants were administered a pregnancy test.

Experimental Protocol: The protocol was devised based on methods from prior publications [17,20,23,25]. The subjects completed three study visits on separate days. Testing visit 1 was for Beverage 1 (Buoy^®^ Hydration Drops, San Diego, CA, USA). Testing visit 2 was for water alone. Testing visit 3 was for Beverage 2 (Nuun^®^ Sport, Seattle, WA, USA). The type of bottled water used (for reconstitution as well as control arm) in all three visits was Kirkland^®^ (Brentwood, TN, USA). Participants refrained from vigorous exercise within 24 h of the study visit. Participants were asked to fast for 10 h through the night prior to each testing day. Upon waking they were asked to empty their bowel and bladder. If desired, they were allowed to consume one 8-ounce cup of coffee or other clear liquid, with the expectation that the participant would be consistent with its consumption with each testing visit.

During each testing visit, subjects presented between 7:00 and 8:00 h, at which time they were asked to empty their bladder again. After resting for five minutes, baseline vitals were taken, including blood pressure and heart rate (Welch Allyn Connex 6000, Skaneateles, New York, NY, USA), weight and bioimpedance (Omron BCM-500, Kyoto, Japan [28,29]). Urinalysis dipstick for protein/blood/glucose (Siemens, Berlin, Germany) and i-STAT (Abbott, Lake County, IL, USA) measurement for creatinine and electrolytes were done on Visit 1 to confirm eligibility. For Visit 2 and Visit 3, the participants were re-interviewed to review any changes in their medical history that would warrant an additional baseline creatinine and blood/protein test to confirm eligibility.

All arms of the study were repeated in every subject. The first arm during the first study visit was Beverage 1, followed by the second study visit for water alone (control), and finally the third study visit for Beverage 2.

The total volume of fluid consumed during each of the three study visits was capped at one liter (1 L) to minimize the risk of water intoxication [3]. The Beverage 1 arm and water-only arm had an identical rate of consumption. For these arms, the entire 1 L fluid was administered in a metered fashion over 4 h. This rate of consumption was chosen to reflect the label recommendation of Beverage 1, to drink the solution in small aliquots throughout the day. The water-only arm followed an identical administration protocol to serve as a true control for Beverage 1. For Beverage 2, the third arm, administration was in a bolus fashion of 1 L over 30 min, based on prior studies showing that bolus consumption of Beverage 2 significantly improved the hydration status compared to water alone [25]. Thus, the Beverage 2 arm served as the positive control.

**Figure 1:**
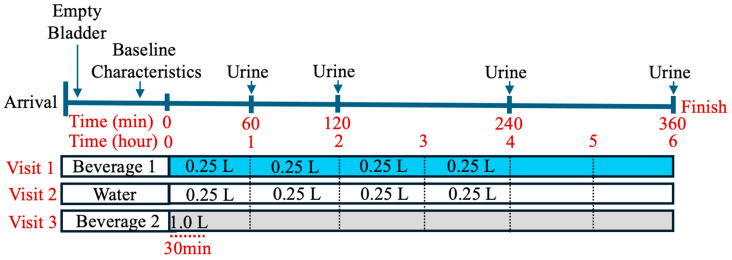
Graphic representing the protocol timeline for each experiment. For Visit 1 (Beverage 1) and Visit 2 (water alone), each subject consumed 6.25% of the total volume of 1 L every 15 min for 4 hours. For Visit 3 (Beverage 2), participants consumed 1 L within the first 30 min. At time 0, the participant began drinking per the above protocol. Urine was collected at 60, 120, 240, and 360 min.

Urine was collected at four specific timepoints during the intervention (60, 120, 240, 360 min) and the mass (grams, g) of the urine was recorded. The mass was assumed equivalent to volume (liters, L). If participants needed to micturate between scheduled collection times, the urine that was collected was recorded and combined with the urine collection of the following timepoint. These urine samples were measured and sent to the University of Pittsburgh Medical Center hospital laboratory to be tested for the following electrolytes: Na^+^, K^+^, Cl^−^ and urine osmolality. No other food or beverage was consumed by the participants during the 6 h of each study visit. After completion of all three portions of the study, participants were compensated USD 300.00 after completion of three visits. See Figure 1 for the protocol timeline.

VISIT 1—BEVERAGE 1 INTERVENTION: Each subject consumed Beverage 1 (Buoy^®^ Hydration Drops, San Diego, CA, USA) at a dose containing 600 mg/L of Na^+^ over 4 h while measuring urine output over 6 h. For Beverage 1, the label recommended dosage is 1.5 mL (50 mg Na^+^ per serving) in multiple servings throughout the day. Therefore, to safely achieve a total dose of 600 mg Na^+^/L (12-fold increase from single dose) we used 18 mL (1.5 mL × 12) of Beverage 1 per 1 L of water consumed. This is equivalent to two servings of the extra-strength Buoy^®^ Rescue Drops. Each subject consumed 6.25% of the total volume every 15 min for four hours. At time 0, the participant began drinking per the above protocol. Urine was collected at 60, 120, 240, and 360 min.

VISIT 2—WATER ALONE CONTROL: Each subject ingested 1 L of Kirkland^®^ bottle water at a rate of 6.25% of the total amount of water every 15 min for four hours. At time 0, the participant began drinking per the above protocol. Urine was collected at 60, 120, 240, and 360 min.

VISIT 3—BEVERAGE 2 INTERVENTION: For Beverage 2, each subject consumed 1 L of water with two dissolved Nuun^®^ Sport Hydration tabs (Nuun, Seattle, WA, USA), containing 600 mg of sodium, over 30 min (2 equal volumes every 15 min) while measuring urine output over 6 h (rate of consumption was based on previously published data by Pence et al. [25]). At time 0, the participants began drinking per the above protocol and were done drinking at 30 min. Urine was collected at 60, 120, 240, and 360 min. A water-only control with a similar rate of 1 L in 30 min was not performed as it was previously published [25].

Calculations: Net fluid balance was calculated as the amount of fluid ingested minus the cumulative urine output at each time point. Participants were considered to be in a positive fluid balance if the net fluid balance was > 0. The beverage hydration index (BHI), developed by Maughan et al., is a method for comparing the short-term hydration efficacy of different beverages [20]. It assesses the hydration potential of a beverage relative to plain water. The BHI is calculated by dividing the cumulative urine volume after ingesting plain water by the cumulative urine volume after consuming the test beverage. Water has a BHI of 1.0. Beverages with a BHI less than 1.0 promote diuresis and are considered ineffective for oral rehydration. Conversely, beverages with a BHI greater than 1.0 limit diuresis and are considered effective for oral rehydration [17]. BHI was calculated at each timepoint for Beverage 1 compared to water since equal rates of Beverage 1 and water-alone were consumed at each timepoint. For the BHI comparison of Beverage 1 to Beverage 2, since the beverages were consumed at different rates the BHI was only calculated at a final timepoint of 360 min.

Statistical Analysis: Data were analyzed using Prism software version 10 (GraphPad Software LLC, Boston, MA, USA) and presented as either mean ± standard deviation (SD) or mean ± 95% confidence interval (CI) of the mean. Prior to analysis, data were checked for normality of distribution. An unpaired two-tailed *t*-test was used for comparisons between two groups. Welch’s was applied for parametric *t*-tests, while Mann–Whitney test was used for non-parametric comparisons. Comparisons between multiple groups and variables were determined using one- or two-way analysis of variance (ANOVA), followed by the appropriate post hoc test, as indicated. *p* values ≤ 0.05 were considered statistically significant. The final sample size was determined a priori using G*Power 3.1 based on previous findings [17,20,23]. Assuming an effect size f^2^(V) = 0.25, α = 0.05; β = 0.2; power = 0.8, requires *n* = 27 participants per beverage.

## 3. Results

### 3.1. Overview

In total, 30 participants successfully completed the study (14 males and 16 females) according to the protocol described (Figure 1). None of these participants reported a problem with beverage consumption, and all beverages were well-tolerated. No patients were excluded from the analysis. The composition of each beverage is in Table 1.

**Table 1:** Beverage composition.

	Water	Beverage 1	Beverage 2
Total Volume	1 L	1 L	1 L
Sodium (mg)	Negligible	600	600
Potassium (mg)	Negligible	120	300
Chloride (mg)	Negligible	960	80
Magnesium (mg)	Negligible	6	50
Calcium (mg)	Negligible	6	26
Calories (kcal/L)	0	0	30
Sugars (g)	0	0	2
Carbohydrates	0	0	8

Based on manufacturer’s nutrition labels. Beverage 1: 18 mL were added to 1 L of water. Beverage 2: 2 tabs were added to 1 L of water.

### 3.2. Baseline Physical Characteristics

Baseline characteristics are shown in Table 2, including age, weight, body mass index (BMI), body composition, blood pressure and heart rate. Several factors were influenced by sex including body weight, body composition (fat versus muscle), and resting metabolism. Factors there were not significantly different between males and females include age, BMI, blood pressure, heart rate, and baseline kidney function.

### 3.3. Beverage 1 Significantly Decreased Urine Volume

**Figure 2:**
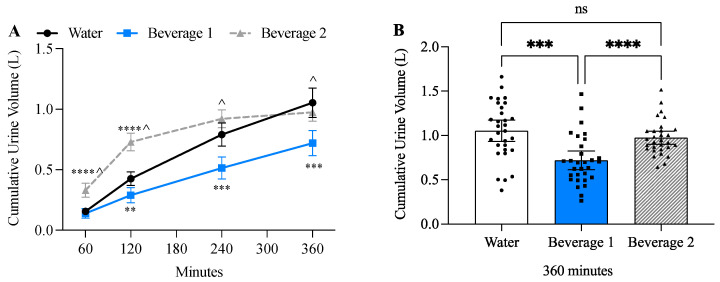
Beverage 1 significantly decreases urine volume. (**A**) Cumulative urine output at each timepoint. For time course experiments error bars represent 95% confidence intervals (CI), and significance is determined by two-way ANOVA at each timepoint with Tukey’s post hoc test. Values significantly different from water are shown as ** *p* ≤ 0.01, *** *p* ≤ 0.001, **** *p* ≤ 0.0001. ^ indicates significant differences (*p* ≤ 0.05) between Beverage 1 and Beverage 2. (**B**) Bar graph showing individual participants’ cumulative urine output after 360 min. Error bars represent 95% CI. Each individual value is graphed to demonstrate standard deviation between beverages. For bar graph experiments significance is determined by one-way ANOVA with Tukey’s post hoc test. *** *p* ≤ 0.001, **** *p* ≤ 0.0001, ns = not significant.

Two-way ANOVA revealed both a time-effect (*p* < 0.0001) and beverage-effect (*p* < 0.0001), and a time × beverage effect (*p* < 0.0001). The rate of urine output (L/minute) reflected the differences in the rate of ingestion (Table 3). Both Beverage 1 and water alone had a slower metered rate of ingestion (1 L/240 min) and urine volume peaked at 240 min and then began to decline, whereas Beverage 2 had a rapid bolus rate of ingestion (1 L/30 min) that was reflected by larger urine volume during the first 120 min that rapidly declined by 240 min (Table 3). Prior studies have shown that when Beverage 2 and water alone were ingested at a similarly rapid rate (1 L/30 min), the rate of urine output was comparable at 60 min, 180 min and 240 min, and the only time Beverage 2 decreased urine output compared to water was at 120 min [25]. In the current study, cumulative urine volume was significantly decreased by Beverage 1 compared to water alone at 120, 240, and 360 min (Figure 2A). Cumulative urine volume was significantly decreased with Beverage 1 compared to Beverage 2 at 60, 120, 240 and 360 min (Figure 2A). Bar graphs revealed that after consuming 1 L of each beverage, cumulative urine volume at 360 min for water was 1.05 L ± SD 0.32, for Beverage 1 it was 0.72 L ± SD 0.28, and for Beverage 2 it was 0.97 L ± SD 0.20 (Figure 2B).

### 3.4. Beverage 1 Significantly Increased Net Fluid Balance

**Figure 3:**
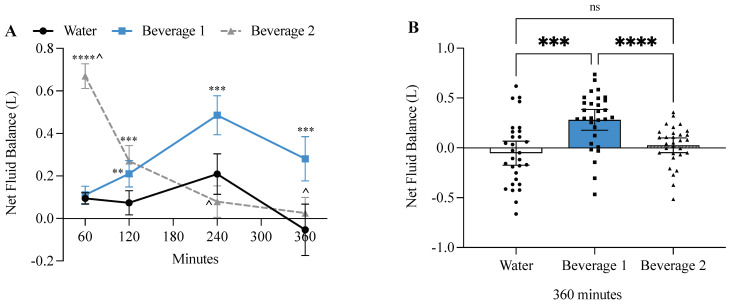
Beverage 1 significantly increases positive fluid balance. (**A**) Net fluid balance was calculated as the amount of fluid ingested minus the cumulative urine output at each time point. For time course experiments error bars represent 95% confidence intervals (CI), and significance is determined by two-way ANOVA at each timepoint with Tukey’s post hoc test. Values significantly different from water are shown as ** *p* ≤ 0.01, *** *p* ≤ 0.001, **** *p* ≤ 0.0001. ^ indicates significant differences (*p* ≤ 0.05) between Beverage 1 and Beverage 2. (**B**) Bar graph showing net fluid balance of individual participants after 360 min. Values > 0 represent positive fluid balance and values < 0 represent negative fluid balance. Each individual value is graphed to demonstrate standard deviation between beverages. Error bars represent 95% CI. For bar graph experiments, significance is determined by one-way ANOVA with Tukey’s post hoc test. *** *p* ≤ 0.001, **** *p* ≤ 0.0001, ns = not significant.

Two-way ANOVA revealed both a time-effect (*p* < 0.0001) and beverage-effect (*p* < 0.0001), and a time × beverage effect (*p* < 0.0001). The rapid ingestion (1 L/30 min) of Beverage 2 maintained a positive fluid balance until 360 min when it approached zero. The slow ingestion (1 L/240 min) of water alone maintained a positive fluid balance until 360 min when it caused a negative fluid balance, and the slow ingestion of Beverage 1 maintained a large positive fluid balance beyond 360 min (Figure 3A). By 360 min, water alone had a fluid balance of −0.05 L ± SD 0.32, Beverage 1 had a fluid balance of +0.28 L ± SD 0.28, and Beverage 2 had a fluid balance of +0.03 L ± SD 0.20 (Figure 3B). Thus, slowly ingested Beverage 1 was at least 10-fold more effective at maintaining a positive fluid balance compared to slowly ingested water alone or rapidly ingested Beverage 2.

### 3.5. Beverage 1 Significantly Increased the Beverage Hydration Index

**Figure 4:**
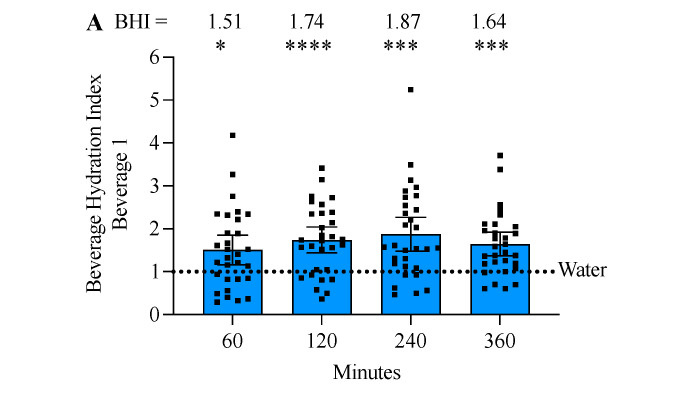

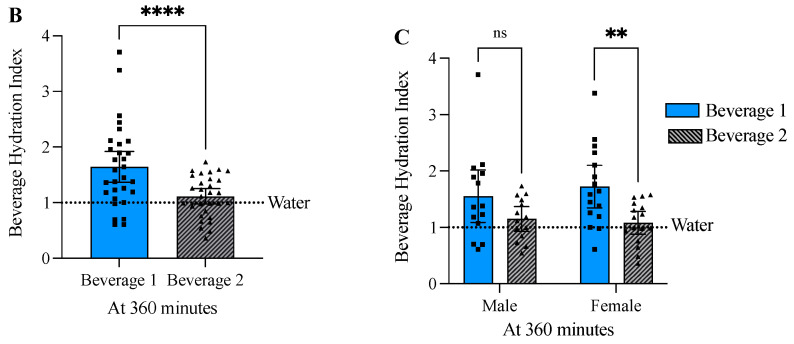
Beverage 1 significantly increases the beverage hydration index (BHI). (**A**) BHI was calculated as the cumulative urine volume with water alone divided by the cumulative urine volume with the intervention beverage. At each timepoint evaluated Beverage 1 significantly increases the BHI compared to water alone. Water has a BHI set to 1.0 (dotted line) and Beverage 1 has a BHI range of 1.51 to 1.87. Error bars represent 95% confidence intervals (CI), and significance is determined by two-way ANOVA at each timepoint with Sidak’s post-test. * *p* ≤ 0.05, *** *p* ≤ 0.001, **** *p* ≤ 0.0001 comparing whether Beverage 1 is significantly different from water-alone. (**B**) Results show calculated BHI of Beverage 1 (1.64 ± SD 0.75) compared to Beverage 2 (1.11 ± SD 0.37) at 360 min. Error bars represent 95% CI and significance is determined by paired two-tailed Wilcoxon *t*-test of Beverage 1 versus Beverage 2. **** *p* ≤ 0.0001. (**C**) Results are grouped by subject-reported sex, the trend for Beverage 1 to have a higher BHI compared to Beverage 2 was similar when separated by sex, but only females reached a significance difference. Error bars represent 95% CI and significance is determined by two-way ANOVA at each timepoint with Sidak’s post-test. ** *p* ≤ 0.01.

Two-way ANOVA revealed both a beverage-effect (*p* < 0.0001) and a time × beverage effect (*p* < 0.047), but not a time-only effect (*p* = 0.077) (Figure 4A). Beverage 1 had a BHI that was significantly greater than water-alone (water = 1.0), with Beverage 1 having a value of 1.51 ± SD 0.93 at 60 min, 1.71 ± SD 0.81 at 120 min, 1.87 ± SD 1.10 at 240 min, and 1.64 ± SD 0.75 at 360 min (Figure 4A). At 360 min, the BHI of Beverage 1 (1.64 ± SD 0.75) was significantly higher than the BHI of bolused Beverage 2 (1.11 ± SD 0.37) despite equal amounts of sodium and volume consumed (Figure 4B). Next, BHI was analyzed based on self-reported sex. The trends were similar for both males and females and were similar to the combined results. However, only the female results reached significance. This was likely due to the small sample size, as males had an *n* = 14 and females had an *n* = 16. Previous studies report no difference in BHI based on sex or body mass, thus combining male and female data is assumed to be appropriate for statistical analysis [17,30].

### 3.6. Beverage 1 Increased Cumulative Urine Osmolality

**Figure 5:**
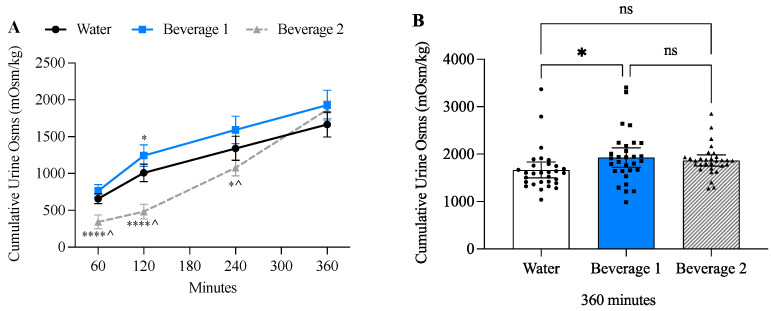
Beverage 1 significantly increases cumulative urine osmolality (osms) compared to water alone. (**A**) Cumulative urine osms. Error bars represent 95% confidence intervals (CI) and significance is determined by two-way ANOVA at each timepoint with Tukey’s multiple comparisons test. * *p* ≤ 0.05, **** *p* ≤ 0.0001 comparing whether water alone is significantly different from Beverage 1 or Beverage 2. ^ indicates significant differences (*p* ≤ 0.05) between Beverage 1 and Beverage 2. (**B**) Bar graphs showing individual participants’ cumulative urine osms at 360 min. There is a significant increase in urine osms in the Beverage 1 group [1928 mOsm/kg ± SD 544] compared to water alone (1666 mOsm/kg ± SD 454). Beverage 2 is not significantly different from either beverage (1866 mOsm/kg ± SD 318). Error bars represent 95% CI. Each individual value is graphed to demonstrate standard deviation between beverages. For bar graphs, significance is determined by one-way ANOVA with Tukey’s post hoc test. * *p* ≤ 0.05, ns = not significant.

Two-way ANOVA revealed both a time-effect (*p* < 0.0001) and beverage-effect (*p* < 0.0001), and a time × beverage effect (*p* < 0.0001) (Figure 5A). After ingesting 0.25 L of water-alone or Beverage 1 over 60 min, urine osmoles (Osms) were 658 milliOsm/kilogram (mOsm/kg) ± SD 180 for water and 764 mOsm/kg ± SD 230 for Beverage 1. Whereas initial urine Osms after ingesting 1 L of Beverage 2 over 30 min were 343 mOsm/kg ± SD 251. Over the next 360 min the water-alone and Beverage 1 similarly decreased urine Osms to a nadir of ~330 mOsms/kg, whereas Beverage 2 decreased to a nadir of ~140 mOsm/kg at 120 min and then increased to 789 mOsm/kg by 360 min (Table 4). The change in urine Osms at each timepoint likely reflects both the rate at which the beverage was consumed and the electrolyte/macronutrient content. When cumulative urine Osms were evaluated at 360 min there was no significant difference between Beverage 1 (1928 mOsm/kg ± SD 544) and Beverage 2 (1866 mOsm/kg ± SD 318); however, the urine Osms of water (1666 mOsm/kg ± SD 454) were significantly lower compared to Beverage 1 (Figure 5B).

### 3.7. Beverage 1 Decreased Cumulative Urine Sodium and Chloride Compared to Water-Alone

**Figure 6:**
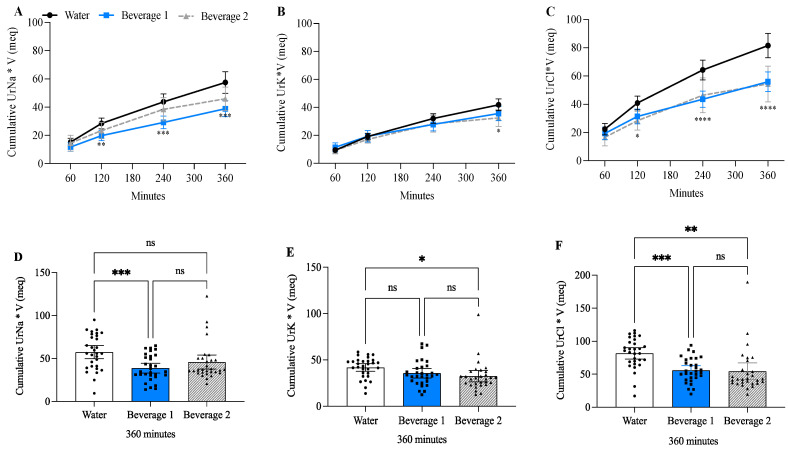
Beverage 1 significantly decreases cumulative urine electrolytes compared to water-alone. (**A**–**C**) Cumulative time course of urine electrolytes normalized to urine volume for total electrolytes excreted in the urine over 360 min. For time course experiments, error bars represent 95% confidence intervals (CI) and significance is determined by two-way ANOVA at each timepoint with Tukey’s multiple comparisons test. * *p* ≤ 0.05, ** *p* ≤ 0.01, *** *p* ≤ 0.001, **** *p* ≤ 0.0001 comparing whether water alone is significantly different from Beverage 1 and Beverage 2. (**D**–**F**) Bar graphs showing individual participants’ cumulative urine electrolytes normalized for volume at 360 min. Beverage 1 had significantly less Na^+^ (39 meq ± SD 15) and Cl^−^ (56 meq ± SD 19) compared to water alone Na^+^ (58 meq ± SD 21) and Cl^−^ (82 meq ± SD 23). Error bars represent 95% CI. Each individual value is shown to demonstrate standard deviation between beverages. For bar graphs significance is determined by one-way ANOVA with Tukey’s post hoc test. * *p* ≤ 0.05, *** *p* ≤ 0.001, ns = not significant. One value for urine K^+^ and urine Cl^−^ were below the limit of detection and excluded from results.

The concentration milliequivalent/L (meq/L) of urine Na^+^, K^+^, and Cl^−^ followed a similar trend to urine Osms, with both water alone and Beverage 1 initially decreasing the electrolyte concentration in the urine and then plateauing after 120 min, whereas Beverage 2 caused an initial decrease that began to rise after 120 min (Table 5). The urine electrolyte concentration was dependent on the rate of beverage ingestion and the volume of urine output. Therefore, to normalize for urine dilution and/or concentration, the urine electrolyte concentrations were normalized to urine volume (V) (Table 5). The excretion of urine electrolytes (meq) tended to be higher with water alone compared to Beverage 1, most notably at 240 min. Cumulative urine Na+ concentration (meq) was consistently higher with water alone compared to Beverage 1 at 120, 240, and 360 min (Figure 6A,D). Cumulative urine K^+^ (meq) was only different between water alone and Beverage 2 at 360 min (Figure 6B,E). Whereas cumulative urine Cl^−^ (meq) was significantly less for Beverage 1 and Beverage 2 compared to water-alone (Figure 6C,F). Beverage 1 at 360 min had significantly less cumulative urinary sodium (Na^+^) (39 meq ± SD 15) and chloride (Cl^−^ (56 meq ± SD 19) compared to water-alone Na^+^ (58 meq ± SD 21) and Cl^−^ (82 meq ± SD 23).

## 4. Discussion

The importance of hydration is often underappreciated and under-researched, despite water being the largest single constituent of the human body and the most essential nutrient for survival [3,31,32]. In this current study we have shown that the ingestion of Beverage 1 improved hydration status in participants at rest based on several markers of hydration including decreased urine volume, positive net fluid balance, and increased beverage hydration index (BHI) [20]. After six hours, Beverage 1 had a 64% increase in the BHI compared to water alone and a 48% increase in BHI compared to Beverage 2. The BHI is a relatively new tool to measure the hydration index [20]. Since its inception in 2016, it has been adapted for several studies to compare the hydrating capabilities beverages ranging from water, milk, coffee, and sports beverages, to oral rehydration solutions [17,20,23,24,25,30]. BHI does not appear to be affected by body mass or sex [30], but is affected by advanced age [23]. For that reason, we limited the age of the study participates to 18–45 years and collectively analyzed the results regardless or sex or body mass.

It has long been known that plain water is ineffective at maintaining proper hydration (euhydration) due to its tendency to reduce plasma osmolality that induce prompt diuresis [12]. In our study, we found that consuming plain water increased urinary electrolyte excretion (meq). While this may seem counterintuitive since water contains no Na^+^, K^+^, or Cl^−^, the findings underscore how water-induced diuresis can negatively affect hydration and increase the risk of electrolyte imbalances. The addition of electrolytes, protein and carbohydrates to water can augment fluid retention and improve hydration. Prior studies have shown that beverages with higher macronutrient and electrolyte content are the most effective at increasing BHI. For example, two hours after bolused consumption, the BHI for skim milk, oral rehydration solution and orange juice was 1.58, 1.54, and 1.39, respectively, compared to water alone [20]. Prior studies have also shown a trend for rapidly ingested Beverage 2 (1 L in 30 min) increased the BHI to 1.2 at two hours, although this finding did not reach statistical significance when analyzed using ANOVA [25].

Sodium is the most common electrolyte studied in hydration beverages. Research has shown that to maximize hydration the sodium concentration should be between 40 and 100 millimole (mmol)/L (920–2300 mg/L), whereas drinks with lower sodium concentrations of 20–30 mmol/L (460–690 mg/L) have inconsistent results and do not always improve markers of hydration [20,23,24,30]. This is important, because most commercially available sports drinks have a sodium concentration ranging from 20 to 30 mmol/L. Compare this to Pedialyte which is closer to 45 mmol/L, or pharmaceutical oral rehydration solutions (ORS) that have a sodium concentration greater than 75 mmol/L [17,24]. A key issue with oral rehydration solutions containing higher sodium contents is that they are unpalatable due to the salty flavor. For this current study we chose a sodium concentration of 26 mmol/L (600 mg/L). Although this is within the lower sodium range, this dose was chosen because it is similar to prior studies looking at the hydration efficacy of Beverage 2 [25], it is closer to the actual daily amount recommended, and avoids the unpalatable flavor of higher sodium doses.

Despite prior studies showing inconsistent results with sodium concentrations between 20 and 30 mmol/L [20,23,24,30], this current study found that Beverage 1 containing 26 mmo/L (600 mg/L) of sodium significantly decreased urine volume, increased net fluid balance, improved the BHI, and increased urine Osms compared to water alone. Moreover, Beverage 1 was more effective at improving markers of hydration than Beverage 2, despite ingestion of identical amounts of fluid (1 L) and sodium (600 mg). There were several factors that may explain these differences including rate of ingestion, carbohydrate content, and chloride concentration.

Prior studies with rapid ingestion rates of 1 L in 30 min demonstrated that negative fluid balance with water typically occurs within 120 min whereas electrolyte-containing beverages extend the time in positive fluid balance [17,20,25]. Only a few studies have evaluated the impact of ingestion rate on hydration status, among these studies there is a consensus that slower metered rates of ingestion are likely to result in more efficient hydration compared to bolus ingestion [5,14,15,16]. This is due to the protective bolus response that occurs with the rapid ingestion of hypotonic fluid [13]. The hypotonic fluid reduces plasma osmolality, and to protect against hyponatremia the body inhibits arginine vasopressin (AVP) release resulting in a prompt diuresis. Despite the paucity of data, it seems clear that large boluses of fluid should be avoided—rather beverages should be consumed over several hours for effective hydration [5].

Traditionally, the beverage hydration index (BHI) is measured after the rapid ingestion of 1 L bolus during the first 30 min of the study (7, 15, 17, 22, 23). To our knowledge only one other study measured BHI with slower fluid ingestion over hours [16]. Thus, as a positive control we included Beverage 2 with rapid ingestion (1 L in 30 min) as it has previously been shown to improve markers of hydration [25]. For this study, Beverage 1 was administered as a slower ingestion over four hours and then compared to the hydration efficacy of water alone also given over four hours or Beverage 2 given as a bolus. We chose to study Beverage 1 over a slower time course because the manufacturer’s label recommends consuming the beverage in multiple servings throughout the day. Based on the BHI after six hours, our results suggest that Beverage 1 with metered ingestion is superior to metered plain water ingestion or to Beverage 2 with bolus ingestion; however, a key limitation in evaluating the rate of ingestion is that we did not directly compare Beverage 1 as a bolus ingestion. Evaluating the rate of ingestion remains an important area of hydration research. Beyond the ingestion rate, there are other variables within the beverages that could affect hydration including macronutrients and electrolytes.

Sports drinks and oral rehydration beverages typically vary in their compositions of carbohydrates, amino acids and electrolyte content. While electrolytes are the most important factor for hydration, carbohydrates and amino acids also play a role [5,17,23,24]. Research has shown that the addition of carbohydrates to beverages can enhance hydration by altering gastric emptying and stimulating glucose–sodium co-transporters which create an osmotic gradient to aid water absorption in the intestines [19,24,33,34]. However, the carbohydrate concentration required for consistent hydration improvements is higher than what is typically found in sports drinks [24]. Moreover, the addition of calories, carbohydrates, and artificial sweeteners can be a barrier for individuals who need to limit excess calories or carbohydrates. Carbohydrates can cause gastrointestinal discomfort [35], and artificial sweeteners such as sorbitol have actually been shown to cause diuresis [36]. In this study, Beverage 1 was free of carbohydrates and artificial sweeteners, while Beverage 2 contained two grams of sugar, eight grams of carbohydrates, and sorbitol.

Potassium levels were also different among the beverages in this study, with Beverage 2 containing more than twice the potassium of Beverage 1. However, this is unlikely to have influenced hydration, as potassium does not enhance fluid retention during post-exercise rehydration when sodium levels are adequate [5]. A striking difference was that Beverage 1 contained over ten times more chloride than Beverage 2 (960 mg or 27 mmol/L in Beverage 1 vs. 80 mg or 2.3 mmol/L in Beverage 2). Chloride, the most abundant anion in the body, is critical for maintaining osmotic pressure, acid–base balance, water movement, and extracellular fluid tonicity, contributing to approximately one-third of extracellular tonicity [37]. Others have postulated that chloride may be important for hydration, but its role remains obscure compared to its sodium counterpart [5,37]. To our knowledge, no studies have specifically examined chloride’s effectiveness in improving hydration compared to alternative anions, such as bicarbonate or citrate [5,34,38]. In commercial sports drinks, bicarbonate and citrate are sometimes substituted for chloride to reduce carbohydrate-induced gastrointestinal symptoms, buffer changes to blood pH during exercise, and enhance palatability [34]. Future studies should focus on isolating the effects of chloride independent from sodium and compare the hydration efficacy to other anions. Additionally, Beverage 1 contains a proprietary blend of trace minerals and B vitamins, though it remains unclear whether these components influence hydration.

A limitation to this study is that we did not power the study to differentiate between males and females even though there are sex-based differences in water handling. Total body water and water intake vary between males and females, with females having lower total body water and total water intake [3]. This is attributed to females having lower metabolic rates, smaller size, and lower fat-free mass [3]. Additionally, males tend to sweat more and have higher electrolyte losses [2]. Males have been found to be more sensitive to arginine vasopressin (AVP) which inhibits diuresis, whereas females tend to have more estrogen and progesterone that enhance water and electrolyte retention [2]. Despite these differences the American College of Sports states that “sex differences in renal water and electrolyte retention are subtle and probably not of consequence” [2]. Reassuringly, previous studies support that BHI measurements are not affected by sex [30]. When we analyzed our BHI results by sex, the trends between males and females remained the same. However, only the female BHI results reached significance, likely due to the results being underpowered for individual sexes.

## 5. Conclusions

While the field of hydration research is growing, in many ways it remains in its infancy and many questions remain unanswered. Much of the current research focuses on the acute effects of hypohydration on athletes and physical performance, yet hydration also has significant implications for overall health [6,31,39]. In the United States, billions of dollars are spent annually on treating dehydration, a preventable condition [11,40,41]. Chronic hypohydration has been associated with increased morbidity and mortality, including conditions such as headaches, kidney stones, exercise-related asthma, heart disease, kidney disease, blood clots, stroke, dental disease, urinary tract infections, bladder and colon cancer, gallstones, mitral valve prolapse, and glaucoma [3,6,39].

Future studies will explore the potential benefits of Beverage 1 for populations beyond healthy and fit individuals. This includes studying its effects on vulnerable groups such as the elderly [6,23], those with gastrointestinal disease [42], and cancer patients [43,44]. Further studies are also needed to examine how ingestion rate and beverage composition influence hydration efficacy. Since hydration is essential for survival, more research is necessary to optimize hydration for improving performance, promoting health, and preventing disease.

## Figures and Tables

**Table 2:** Baseline physical characteristics of participants at 1st visit.

	Male ± SD (*n* = 14)	Female ± SD (*n* = 16)	Combined ± SD (*n* = 30)
Age (years)	31.6 (±6.6)	28.3 (±4.3)	29.9 (±5.6)
Body Weight (kg)	88.96 (±12.8)	76.9 (±18.5) *	82.6 (±16.9)
BMI	27.1 (±3.8)	28.1 (±7.6)	27.7 (±6.1)
Body Fat %	23.4 (±8.6)	33.7 (±9.3) **	28.7 (±10.2)
Skeletal Muscle %	36.6 (±6.0)	28.6 (±4.8) ***	32.5 (±6.7)
Resting Metabolism (kcal)	1868 (±166)	1535 (±199) ***	1695 (±248)
Visceral Fat	7.4 (±3.5)	6.5 (±2.8)	7.0 (±3.2)
Systolic Blood Pressure	120.4 (±3.4)	119.7 (±2.9)	120.0 (±3.1)
Diastolic Blood Pressure	77.1 (±2.0)	77.3 (±1.8)	77.2 (±1.9)
Heart Rate	77.9 (±7.5)	80.0 (±11.3)	79.0 (±9.6)
Serum Creatinine	0.94 (±0.09)	0.97 (±0.12)	0.95 (±0.11)

Statistical significance between male and female was determined using unpaired two-tailed *t*-test described in the methods. Female values significantly different from male values are shown as * *p* ≤ 0.05, ** *p* ≤ 0.01, *** *p* ≤ 0.001. Sex was self-reported.

**Table 3:** Urine volume (L) collected at each time point.

	Water (L) (±SD) (*n* = 30)	Beverage 1 (L) (±SD) (*n* = 30)	Beverage 2 (L) (±SD) (*n* = 30)
60 min	0.156 (±0.074)	0.139 (±0.109)	0.330 (±0.155) ****^
120 min	0.270 (±0.139)	0.151 (±0.084) ***	0.399 (±0.105) ***^
240 min	0.365 (±0.170)	0.225 (±0.143) **	0.191 (±0.172) ***
360 min	0.262 (±0.125)	0.205 (±0.065)	0.054 (±0.038) ****^

L = liters. Statistical significance between beverages at each time point was determined by two-way ANOVA with Tukey’s post hoc test. Values significantly different from water are shown as ** *p* ≤ 0.01, *** *p* ≤ 0.001, **** *p* ≤ 0.0001. ^ indicates significant differences (*p* ≤ 0.05) between Beverage 1 and Beverage 2.

**Table 4:** Urine Osms (mOsm/kg) collected at each time point.

	Water (±SD) (*n* = 30)	Beverage 1 (±SD) (*n* = 30)	Beverage 2 (±SD) (*n* = 30)
60 min	658 (±180)	764 (±230)	343 (±251) ****^
120 min	352 (±179)	479 (±210) *	140 (±40) ****^
240 min	331 (±164)	351 (±181)	595 (±168) ****^
360 min	325 (±68)	335 (±91)	789 (±111) ****^

Statistical significance between beverages at each time point was determined by two-way ANOVA with Tukey’s post hoc test. Values significantly different from water are shown as * *p* ≤ 0.05, **** *p* ≤ 0.0001. ^ indicates significant differences (*p* ≤ 0.05) between Beverage 1 and Beverage 2.

**Table 5:** The effect of beverage on urine electrolytes at individual time points.

	Urine Na^+^ Concentration (meq/L)			Urine Na^+^ Normalized to Urine Volume (UNa·V) (meq)		
	Water (± SD)	Beverage 1 (± SD)	Beverage 2 (± SD)	Water (± SD)	Beverage 1 (± SD)	Beverage 2 (± SD)
60 min	103.7 (±37.0)	92.2 (±34.0)	48.5 (±47.8) ****^	15.2 (±7.3)	11.8 (±8.0)	14.5 (±14.9)
120 min	55.2 (±33.5)	63.7 (±42.9)	22.6 (±7.5) ****^	13.2 (±7.5)	8.0 (±4.1) **	8.9 (±3.6) *
240 min	48.5 (±28.0)	46.3 (±35.2)	87.3 (±35.2) ****^	15.4 (±6.5)	9.4 (±7.5) **	15.1 (±14.2)
360 min	49.1 (±20.3)	46.4 (±19.4)	142.7 (±34.9) ****^	13.7 (±10.2)	9.7 (±5.1)	7.4 (±4.6) *
	Urine K^+^ concentration (meq/L)			Urine K^+^ normalized to urine volume (UK·V) (meq)		
	Water (±SD)	Beverage 1 (±SD)	Beverage 2 (±SD)	Water (±SD)	Beverage 1 (±SD)	Beverage 2 (±SD)
60 min	65.2 (±16.3)	91.1 (±45.4) *	29.5 (±19.3) ****^	9.5 (±3.9)	11.5 (±8.6)	9.2 (±6.3)
120 min	41.7 (±20.9)	57.7 (±26.7) *	19.0 (±4.6) ****^	9.7 (±4.8)	7.9 (±4.7)	7.6 (±2.8) *
240 min	41.1 (±19.9)	42.8 (±16.2)	61.7 (±31.2) *^	12.9 (±4.6)	8.4 (±3.8) ***	11.1 (±13.4)
360 min	37.3 (±11.2)	39.4 (±13.1)	78.3 (±20.6) ****^	9.8 (±5.1)	7.8 (±3.0)	4.0 (±2.4) ****^
	Urine Cl^−^ concentration (meq/L)			Urine Cl^−^ normalized to urine volume (UCl·V) (meq)		
	Water (±SD)	Beverage 1 (±SD)	Beverage 2 (±SD)	Water (±SD)	Beverage 1 (±SD)	Beverage 2 (±SD)
60 min	149.8 (±43.4)	154.7 (±54.9)	56.4 (±55.7) ****^	22.4 (±10.7)	19.3 (±12.0)	16.6 (±16.5)
120 min	78.7 (±42.3)	92.0 (±44.4)	28.6 (±9.9) ****^	18.5 (±8.5)	12.1 (±5.0) **	11.3 (±4.6) ***
240 min	74.0 (±39.7)	61.9 (±36.5)	96.6 (±36.3) ^	23.4 (±8.9)	12.1 (±6.9) ****	18.5 (±24.1)
360 min	63.9 (±20.9)	60.3 (±19.5)	152.8 (±35.3) ****^	17.2 (±10.6)	12.5 (±5.7)	7.8 (±4.4) ***^

*n* = 30 urine samples per beverage. Statistical significance between beverages at each time point was determined by two-way ANOVA with Tukey’s post hoc test. Values significantly different from water are shown as * *p* ≤ 0.01 ** *p* ≤ 0.01, *** *p* ≤ 0.001, **** *p* ≤ 0.0001. ^ indicates significant differences (*p* ≤ 0.05) between Beverage 1 and Beverage 2.

## Data Availability

The datasets generated and/or analyzed during the current study are not publicly available but are available from the corresponding author on reasonable request via email, and will be shared via secure platform, after approval by corresponding author, with a signed data access agreement.
